# Long-Term Wastewater Surveillance for SARS-CoV-2: One-Year Study in Brazil

**DOI:** 10.3390/v14112333

**Published:** 2022-10-25

**Authors:** Renan Moura Martins, Tamara Carvalho, Cintia Bittar, Daniela Muller Quevedo, Rafael Nava Miceli, Mauricio Lacerda Nogueira, Helena Lage Ferreira, Paulo Inácio Costa, João Pessoa Araújo, Fernando Rosado Spilki, Paula Rahal, Marilia Freitas Calmon

**Affiliations:** 1 Laboratory of Genomic Studies, Institute of Biosciences, Letters and Exact Sciences (IBILCE), São Paulo State University (UNESP), São José do Rio Preto 15054-000, SP, Brazil; 2 Institute of Exact and Technological Sciences (ICET), University Feevale, Novo Hamburgo 93525-075, RS, Brazil; 3 SeMAE-Autonomous Municipal Water and Sewage Service, São José do Rio Preto 15048-000, SP, Brazil; 4 Virology Research Laboratory (LPV), Faculty of Medicine of São José do Rio Preto (FAMERP), São José do Rio Preto 15090-000, SP, Brazil; 5 Applied Preventive Veterinary Medicine Laboratory, Department of Veterinary Medicine, Faculty of Animal Science and Food Engineering (FZEA), University of São Paulo (USP), Pirassununga 13635-900, SP, Brazil; 6 Department of Clinical Analysis, School of Pharmaceutical Sciences, São Paulo State University (UNESP), Araraquara 14801-360, SP, Brazil; 7 Biotechnology Institute, São Paulo State University (UNESP), Botucatu 18607-440, SP, Brazil; 8 Molecular Microbiology Laboratory, University Feevale, Novo Hamburgo 93525-075, RS, Brazil

**Keywords:** SARS-CoV-2, wastewater, qRT-PCR, epidemiology

## Abstract

Wastewater-based epidemiology (WBE) is a tool involving the analysis of wastewater for chemicals and pathogens at the community level. WBE has been shown to be an effective surveillance system for SARS-CoV-2, providing an early-warning-detection system for disease prevalence in the community via the detection of genetic materials in the wastewater. In numerous nation-states, studies have indicated the presence of SARS-CoV-2 in wastewater. Herein, we report the primary time-course monitoring of SARS-CoV-2 RNA in wastewater samples in São José do Rio Preto-SP/Brazil in order to explain the dynamics of the presence of SARS-CoV-2 RNA during one year of the SARS-CoV-2 pandemic and analyze possible relationships with other environmental parameters. We performed RNA quantification of SARS-CoV-2 by RT-qPCR using N1 and N2 targets. The proportion of positive samples for every target resulted in 100% and 96.6% for N1 and N2, respectively. A mean lag of -5 days is observed between the wastewater signal and the new SARS-CoV-2-positive cases reported. A correlation was found between the air and wastewater temperatures and therefore between the SARS-CoV-2 viral titers for N1 and N2 targets. We also observed a correlation between SARS-CoV-2 viral titers and media wastewater flow for the N1 target. In addition, we observed higher viral genome copies within the wastewater samples collected on non-rainy days for the N1 target. Thus, we propose that, based on our results, monitoring raw wastewater may be a broadly applicable strategy that might contribute to resolving the pressing problem of insufficient diagnostic testing; it may represent an inexpensive and early-warning method for future COVID-19 outbreaks, mainly in lower- and middle-income countries.

## 1. Introduction

The SARS-CoV-2 pandemic has global consequences on public health, the economy, and society. The first confirmed SARS-CoV-2 case in the São José do Rio Preto metropolitan area occurred on March 13, 2020, when the country had counted only 34 confirmed cases. This means that this city was among the first in the national territory to identify the transmission of the virus [1]. Peaks in SARS-CoV-2-positive cases in São José do Rio Preto were seen in July and August 2020, November 2020, and January 2021, with the highest peaks in March and April 2021. The prevalent variant diversified over time, with seven lineages detected in January and February 2021. An increase in the prevalence of the Gamma lineage was observed from January 2021, replacing Zeta and other lineages [2].

Despite COVID-19 being a respiratory disease, SARS-CoV-2-viable virus and viral RNA are present in bodily excreta such as saliva, sputum, and feces, which are discarded in wastewater [3]; the viral shedding can vary among individuals, with means of 14–21 days [4,5]. The main route of SARS-CoV-2 transmission is inhalation via person-to-person aerosols/droplets [6]. However, evidence indicates the need for deeper comprehension of the wastewater as a possible source of virus in epidemiological studies [7].

Wastewater monitoring has been used as a strategy of viral disease surveillance through the detection of genetic material in wastewater [8,9,10]. Hence, monitoring of SARS-CoV-2 in sewage is considered a sensitive method to analyze the dissemination of the virus in the population and provide early detection of the virus in the community [11].

Recent reports indicated the presence of SARS-CoV-2 in wastewater in several nation-states [12,13,14,15]. These reports highlight the requirement for effective environmental surveillance. Wastewater-based epidemiology (WBE) as a public health surveillance tool involves the screening of wastewater at the community level and serves as an early-warning system [16]. This methodology is a cost-effective way to assess the dissemination of the infection by monitoring the viral load in the wastewater that contains feces excreted from both symptomatic and asymptomatic individuals, and, therefore, reduces the time and resources spent on clinical surveillance [13,17].

Herein, we report the first time-course monitoring of SARS-CoV-2 RNA in wastewater samples in São José do Rio Preto-SP/Brazil in order to describe the dynamics of the presence of SARS-CoV-2 RNA during one year of the SARS-CoV-2 pandemic and to analyze a possible relationship with several environmental parameters. 

## 2. Methodology

### 2.1. Sampling

Twenty-four-hour composite samples of raw wastewater were collected three times per week for one year (from 15 July 2020 to 15 July 2021) from the Preliminary Treatment unit of the Sewage Treatment Plant (STP) Rio Preto. This plant processes 98–99% of all sewage generated in the municipality, with a total flow of approximately 100,826 m^3^/day and serving approximately 470,000 inhabitants. The samples were collected in a HACH Sigma SD900 AWRS refrigerated automatic sampler (HACH, Loveland, CO, USA), with a sampling program adjusted to the input flow, with the collection of 1 sample for every 3000 m^3^ processed by the plant (equivalent to approx. 36 sampling events/day). Once collected, the samples were kept at below 4 °C and sent to STP Rio Preto Laboratories for decantation for 10 min, followed by vacuum filtration on 47 mm glass fiber membranes with a 1 µm pore size (PALL CORPORATION, Ann Harbor, MI, USA). After filtration, on the day following sampling, 200 mL of each sample was transported to the São Paulo State University Laboratory in glass flasks on ice. Upon receipt, the samples were concentrated by high-speed centrifugation at 40,000× *g* for 3 h at 4 °C using the ultracentrifuge (Beckman Coulter, Indianapolis, IN, USA). Viral pellets were resuspended in 1 mL of DEPC-treated water (Sigma-Aldrich, Saint Louis, MI, USA) and stored at −150 °C. Samples were processed using Class II biological safety cabinets, and standard precautions were applied.

### 2.2. RNA Extraction and Quantitative PCR

RNA extraction of 200 μL of each sample was performed using Trizol reagent (Thermo Fisher Scientific, Waltham, MA, USA), according to the manufacturer’s instructions. RNA was eluted in 30 µL of DEPC water (Sigma-Aldrich, Saint Louis, MI, USA) and stored at −80 °C.

SARS-CoV-2 RNA was detected and quantified using qPCRBIO 1-step Go (PCR Biosystems, London, UK) and the RT-qPCR diagnostic-panel assays were validated by the US Centers for Disease Control and Prevention [18]. The version of the kit with two sets of oligonucleotide primers and probes was used to target two different SARS-CoV-2 regions of the nucleocapsid (N) gene (N1 and N2). The sets of primers and probe (2019-nCoV RUO Kit) (Integrated DNA Technologies, Leuven, Belgium) as well as the positive control (2019-nCoV_N_Positive Control, 2 × 10^5^ genome copies/μL (gc/μL)) were provided by IDT (Integrated DNA Technologies, Leuven, Belgium). A measure of 5 µL of RNA was added to the Reaction mix (15 μL), consisting of 7.5 μL 2X qPCRBIO 1-step Go mix, 0.15 μL RTase Go (PCR Biosystems, London, UK), and 1.13 μL for each set of primers and probe. The thermal cycling conditions were as RT at 50 °C for 10 min, preheating at 95 °C for 2 min and 40 cycles of amplification at 95 °C for 5 s and 60 °C for 30 s. All amplifications were conducted on a QuantStudio 12 K Flex instrument (Applied Biosystems, Foster City, CA, USA). Each RNA was analyzed in triplicate for each primer set and every RT-qPCR assay included negative (nuclease-free water) and positive controls.

Serial tenfold dilutions of the standard plasmid of SARS-CoV-2, obtained from IDT (Integrated DNA Technologies, Leuven, Belgium), were used to produce standard curves. Molecular-biology-grade water was used as a non-template control. The amplification efficiencies (*E*) were calculated based on the equation: *E* = 10^(−1/slope)^ − 1. Negative and positive controls were included in each RT-qPCR run and all RT-qPCR assays were performed in triplicate. Samples were discarded if they did not meet the following conditions: (i) standard curves with R^2^ ≥ 0.95; (ii) copies/reaction in linear dynamic range of the curve; (iii) primer efficiency between 90% and 130% [19].

### 2.3. Detection Limit and qPCR Inhibition Control

The detection limit for the assay was estimated by spiking serial 10-fold dilutions of SARS-CoV-2 synthetic plasmid in wastewater samples (*n* = 5). After the addition of known concentrations of the virus, the spiked water samples were analyzed by real-time RT-qPCR. The lowest concentration of the viruses in the spiked water samples that gave a positive result in the RT-qPCR was taken as an estimate of the detection limit. Thus, the limit of detection of the RT-qPCR assay was determined for N1 and N2 gene regions by determining the number of copies per reaction, which corresponds to a detection rate of ≥ 95% (<5% false negatives), as recommended by the MIQE guidelines [19].

To check for the presence of inhibitors in our samples, all samples testing SARS-CoV-2 N2 negative (*n* = 5), twenty samples testing SARS-CoV-2 N1- and N2-positive, as well as sterile water controls (in triplicate), were inoculated in parallel with a known amount (10^5^ Genome Copies) of SARS-CoV-2 synthetic plasmid containing N region of SARS-CoV-2 (Integrated DNA Technologies, Leuven, Belgium) and tested using a previously described RT-qPCR assay targeting the N region. The SARS-CoV-2 plasmid was also analyzed by PCR, without prior mixing with RNA from the samples. An increase in the threshold cycles (Ct), after addition of the nucleic acid extracts by more than 1 cycle, was considered to indicate inhibition. All qPCR reactions were carried out in triplicate on a QuantStudio 12 K Flex instrument (Applied Biosystems, Foster City, CA, USA) [20].

### 2.4. Data Analysis

Data were analyzed using descriptive statistics through measures of central tendency (mean and median) and measures of dispersion (standard deviation). To test the distribution of variables, the Kolmogorov–Smirnov test was applied. The data did not show normal distribution, so non-parametric tests were applied for statistical inference. Variables N1 and N2 were normalized with reference to flow, as suggested by Nagarkar et al. (2022) [21]. To identify the correlation between N1 and N2, SARS-CoV-2-positive clinical cases, and hydrological and meteorological data, the instantaneous correlation was initially evaluated (lag = 0). The cross-correlation function was also used to assess the correlation between N1 and N2 with positive clinical SARS-CoV-2 cases for various lags; each lag represents two/three days, since the wastewater sample was collected every two/three days (three times per week).

To compare N1 and N2 between rainy and non-rainy days, the Mann–Whitney test was applied. Statistical inference was applied considering a significance level of 5%. The software used for statistical analysis was SPSS V24.

## 3. Results and Discussion

**Figure 1:**
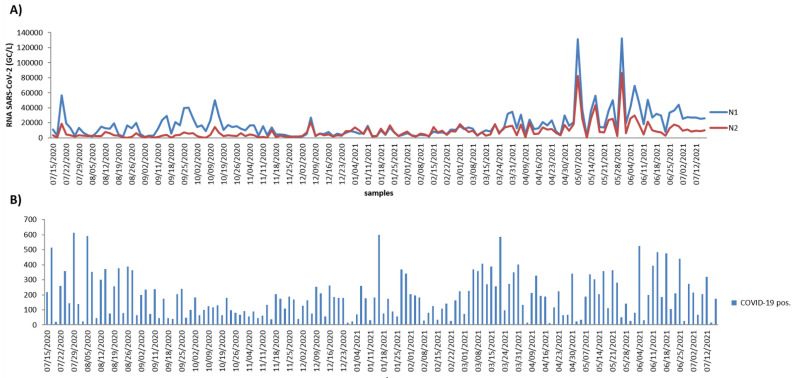
SARS-CoV-2 RNA quantification of the wastewater from São José do Rio Preto and SARS-CoV-2-positive cases in São José do Rio Preto. (**A**) SARS-CoV-2 RNA quantification in the wastewater samples in São José do Rio Preto during one year using the N1 target (blue line) and the N2 target (red line) and (**B**) SARS-CoV-2-positive cases in São José do Rio Preto diagnosed during one year.

SARS-CoV-2 virus RNA was measured by RT–qPCR using the same N1 and N2 primer sets. The percentage of positive samples for each target resulted in 100% and 96.6%, for N1 and N2, respectively. Virus RNA copies ranged from 1 × 10^3^ copies/L to 1.3 × 10^5^ copies/L for N1 target of raw sewage and from 0 to 8.6 × 10^4^ copies/L for the N2 target of raw sewage (Table 1). All qRT–PCR concentration threshold (Ct) values were below 40, and 97% of all samples had a Ct value less than 38 for the N1 primer and 97.3% for the N2 primer. It is interesting to observe that N1 and N2 signals present a similar tendency in wastewater samples (Figure 1), as also observed by long-term surveillance of wastewater in France [22]. The concentrations of SARS-CoV-2 RNA (1 × 10^3^ – 1.3 × 10^5^ copies/L) in wastewater samples in this study are in agreement with studies from the USA [23,24,25], South Africa [26], and India [27]. Interestingly, the concentration of SARS-CoV-2 RNA in this study was higher than that reported in Haramoto et al. in Japan [28], Hasan et al. in Saudi Arabia [29], and Ahmed et al. in Australia [13], but lower than those reported by Balboa et al. in Spain [30]. This could be attributable to differences in abundance of SARS-CoV-2 in wastewater due to the pandemic level in the population; the number of SARS-CoV-2 RNA gene copies in wastewater is correlated with the total number of SARS-CoV-2-positive cases in a community, providing an indication of the total burden of disease on that population beyond merely those individuals identified through SARS-CoV-2 testing. In addition, different sampling methodologies for viral RNA detection and viral quantification analysis may result in variation in the amount of SARS-CoV-2 detected in wastewater.

The in vitro-transcribed viral RNA was detected to a limit of detection (LOD) of 1000 copies/L in both the N1 and N2 RT-qPCR assays and the standard curves demonstrate good linearity for RT-qPCR in a range from 1000 to 10^8^ copies/L for N1 and N2 primers. The LOD in this study was similar to that observed by Ahmed et al. [31]. The linearity and LOD are important parameters according to MIQE guidelines [19] and, although RT-qPCR is a sensitive and specific technique for viral quantification, the reproducibility and reliability of the assay are important parameters.

**Figure 2:**
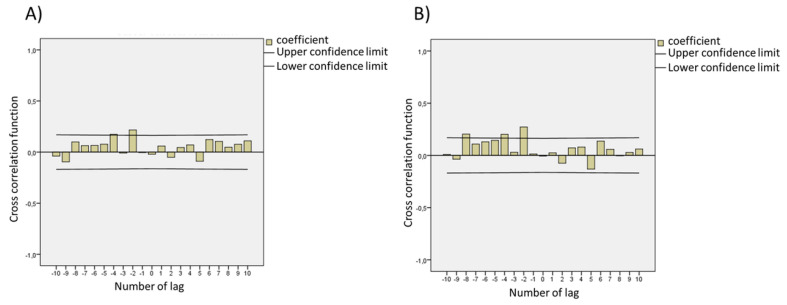
Modeling reveals that the time delay between clinical case reporting and wastewater data changes over the course of the pandemic using (**A**) SARS-CoV-2 N1 target and (**B**) SARS-CoV-2 N2 target. The confidence limit applied was 95%.

All 150 measures traced the increase and decrease in SARS-CoV-2 infections during the 52-week period studied and we analyzed the instant correlation between SARS-CoV-2 and environmental parameters as well as SARS-CoV-2-positive clinical cases. The time-course monitoring of viral load in wastewater displayed two peaks: one on 7 May 2021 and one on 31 May 2021 for both primers. These were followed by a slight decrease (1-log reduction in average). The peaks of the SARS-CoV-2-positive clinical cases on these days were not observed, presenting some peaks on different days such as, for example, one on 29 July 2020 and another on 15 January 2021. A significant relation of viral titers with SARS-CoV-2-positive clinical cases was not observed when instant correlation was applied (Figure 2, Lag 0). We also analyzed the correlation of viral titers with SARS-CoV-2-positive clinical cases; applying a cross correlation of 20 lags allowed an estimation of relationships between viral time-series results and the reported population-testing data. We used the wastewater sample date as a reference and determined the lag time of clinical cases in relation to the wastewater’s SARS-CoV-2 RNA concentration. A negative lag means that the detection of SARS-CoV-2 RNA in the wastewater was observed before the SARS-CoV-2-positive cases, and a positive number indicates that wastewater follows the incidence rate (i.e., changed after). An average lag of −5 days was observed between the detection of SARS-CoV-2 RNA in the wastewater and the new SARS-CoV-2-positive cases reported (Figure 2). This result corroborates a study from the USA that observed a lag of 7–9 days between SARS-CoV-2 RNA concentration and SARS-CoV-2-positive cases reported [32]. In addition, there are reports that SARS-CoV-2 was detected in wastewater earlier than medical reporting by 2 days in Canada [33] and by 2–4 days in the USA [34]. Interestingly, based on clinical tests, Kumar et al. realized the seriousness of the pandemic situation in India 1–2 weeks before the official reports [35]; these time lags between the wastewater signal and the SARS-CoV-2-positive reported cases is coherent with the average 4–5 day incubation period from SARS-CoV-2 infection to symptom onset. On the other hand, a study from Greece applied several statistical models to determine the association between SARS-CoV-2 RNA concentrations in sewage and data for 7 days of cumulative cases and observed no clear evidence that wastewater measurements can anticipate SARS-CoV-2-positive reported cases [36]. These differences in results are observed because wastewater-based epidemiology depends on the viral shedding dynamics relative to symptom onset and the divergence between detection of SARS-CoV-2 RNA in the wastewater and clinical data reporting [37]. The majority of methods for tracking SARS-CoV-2 infection primarily rely on clinical test results, but this process involves intrinsic delays that preclude real-time tracking of the outbreak; to overcome this issue, we compared our wastewater surveillance data with SARS-CoV-2 clinical positive cases collected from the same day.

Alterations in the environment can influence the viral viability and temperature was identified as a significant variable controlling microbial decay and the persistence of viruses in environmental systems. Seasonal variations in wastewater temperature during a year differ around the world. In addition, seasonal changes in air and soil temperature influence the transference of heat between wastewater and the surrounding environment [38]. A negative correlation was found between the air and wastewater temperatures and the SARS-CoV-2 viral titers for N1 (air: R = −0.361, *p* < 0.01; wastewater: R = −0.375, *p* < 0.01) and N2 (air: R = −0.3000, *p* < 0.01; wastewater: R = −0.185, *p* < 0.05) primers (Table 2). Our results corroborate a mixed-model effect, which showed that temperature levels were significantly correlated with SARS-CoV-2 gene recovery [39]. It was reported that with increasing temperature, the incidence of the disease decreased in most of the cities analyzed [40]. One study reported that, frequently, SARS-CoV-2 tends to be rapidly inactivated at high temperatures instead of at low temperatures [41]. Kampf et al. conducted a study in which it was observed that for a 1 °C increase in the minimum ambient air temperature, the cumulative number of cases decreased by almost 1%. However, at ambient temperatures higher than 30 °C, the duration of SARS-CoV-2 detection is reduced [42]. Henwood et al. (2020) suggested that a temperature of 56 °C for 90 min or a temperature of 67 °C for 60 min can inactivate SARS-CoV-2 [43]. Therefore, the persistence of the virus is insignificant and mostly gets destroyed in high ambient and water temperatures.

We also observed a negative correlation between SARS-CoV-2 viral titers and media wastewater flow for the N1 primer (R = −0.298; *p* < 0.01). The SARS-CoV-2 RNA can be influenced by sample location and type, as well as the time of collection. This may change depending on where and when the sample is collected in the wastewater treatment facility because of the differences in the sewage compositions and dilutions. The SARS-CoV-2 RNA detection may also be influenced by sampling type (grab versus composite), and the time of day when it is collected [44]. Thus, it is suggested that a time composite sampling mode may under- or overestimate viral quantification when the flow varies, and when flow and viral titers are positively or negatively correlated [45].

In addition, there is a significant difference in the SARS-CoV-2 viral titers when comparing rainy days (*n* = 40) with non-rainy days (*n* = 110) (U = 1595.00; *p* = 0.010), i.e., there are more viral genome copies in the wastewater samples collected on non-rainy days for the N1 primer (Md_rain_=5855.00; Md_non-rain_=13,603.00) (Table 3). Lazuka et al. observed that SARS-CoV-2 RNA quantification appeared to be strongly influenced by rainfall events as SARS-CoV-2 RNA quantification decreased on rainy days. However, we need to consider that the virus keeps circulating in the population on rainy days and a decrease in concentration or the absence of SARS-CoV-2 RNA detection must be considered with caution when rain events occur [22].

## 4. Conclusions

In conclusion, SARS-CoV-2 RNA concentrations in wastewater treatment plant influent (raw sewage) were analyzed over one year and compared with environmental and populational parameters. Monitoring raw sewage is a broadly applicable strategy that can contribute to the problem of insufficient diagnostic testing and provide an early-warning tool for future SARS-CoV-2 outbreaks, mainly in lower- and middle-income countries. In addition, wastewater-based epidemiology can be used as a tool to aid decision making in the SARS-CoV-2 post-isolation phase and reinstatement of isolation facing a seasonal re-emergence.

## Figures and Tables

**Table 1:** Parameters of wastewater samples collected and SARS-CoV-2 RNA quantification using N1 and N2 targets.

Parameters	Minimum	Maximum	Average	Standard Deviation	Median (Md)
N1 RNA copies: normalized to flow	10.7 × 10^10^	1333.2 × 10^10^	169.6 × 10^10^	183.7 × 10^10^	125.9 × 10^10^
N2 RNA copies: normalized to flow	0.00	837.4 × 10^10^	85.7 × 10^10^	110.6 × 10^10^	54.4 × 10^10^
SARS-CoV-2-positive cases	12.00	613.00	188.34	140.11	174
Air temperature (°C)	0.00	34.30	24.41	3.70	24.5
Average flow (L/s)	1019.00	1394.00	1169.55	71.62	1162
Total flow (m^3^/day)	87,876.00	121,542.00	100,826.41	6150.29	100,067
pH	7.04	7.81	7.51	0.11	7.5
Wastewater temperature (°C)	18.00	30.80	26.03	2.37	26.4
Chemical oxygen demand (mg/L)	411.00	2039.00	725.60	283.56	622.5

**Table 2:** Spearman non-parametric correlation of environmental and wastewater parameters and SARS-CoV-2 N1 and N2 targets. Correlation coefficients: high—R > = 0.6; modest—0.3 < R < 0.6; low—R < 0.3 [46,47].

		N1	N2
SARS-CoV-2-Positive cases	Correlation Coefficient	−0.033	0.000
	Sig. (bilateral)	0.69	0.999
	N	150	150
Air Temperature (°C)	Correlation Coefficient	−0.361 ^**^	−0.300 ^**^
	Sig. (bilateral)	>0.001	>0.001
	N	150	150
pH	Correlation Coefficient	0.215 ^**^	0.281 ^**^
	Sig. (bilateral)	0.008	>0.001
	N	150	150
Wastewater Temperature (°C)	Correlation Coefficient	−0.375 ^**^	−0.185 ^*^
	Sig. (bilateral)	>0.001	0.024
	N	150	150
Average Flow (L/s)	Correlation Coefficient	−0.298 ^**^	0.063
	Sig. (bilateral)	>0.001	0.447
	N	150	150

* 5% significance; ** 1% significance.

**Table 3:** Descriptive analysis of the parameters (Flow, N1 and N2 copies) related to periods with and without rainfall.

Descriptive Statistics									
Rainfall		N	Mean	Standard Deviation	Minimum	Maximum	Percentiles		
							25thg.	50th (Median)	75th
0	Total Flow	110	99,792.7	5139.1	87,876.0	120,384.0	96,797.7	99,877.5	102,610.7
	CopiesN1	110	19,518.2	21,077.7	1076.0	132,348.0	6112.0	13,603.0	26,180.5
	CopiesN2	110	9283.9	12,740.1	0	86,292.0	2422.8	5855.0	11,384.5
1	Total Flow	40	103,668.9	7707.1	91,892.0	121,542.0	98,543.7	101,957.0	109,617.5
	CopiesN1	40	10,719.9	8621.5	1400.0	36,542.0	4246.5	7990.0	15,051.0
	CopiesN2	40	6653.4	5646.2	1352.0	23,572.0	2756.0	4482.0	8792.5
